# Evaluating spatial accessibility to COVID-19 vaccine resources in diversely populated counties in the United States

**DOI:** 10.3389/fpubh.2022.895538

**Published:** 2022-07-25

**Authors:** Feng Qi, Daniela Barragan, Maverick Garcia Rodriguez, Jiongcheng Lu

**Affiliations:** ^1^School of Environmental and Sustainability Sciences, Kean University, Union, NJ, United States; ^2^Gobal Business School for Health, University College London, London, United Kingdom

**Keywords:** COVID-19, vaccine, accessibility, equity, disparity

## Abstract

This study examines the accessibility to COVID-19 vaccination resources in two counties surrounding Newark, NJ in the New York Metropolitan Area, United States. The study area represents diverse population makeups. COVID-19 vaccines were made available by different types of vaccination sites including county mass vaccination sites, medical facilities and pharmacies, and a FEMA community vaccination center in spring 2021. We used the two-step floating catchment area method to measure accessibility and calculated the average accessibility scores of different population groups. We examined the patterns and tested the significance of the differences in accessibility across population groups. The results showed clear spatial heterogeneity in the accessibility to vaccine resources with the existing infrastructure (medical/pharmacy vaccine sites). Accessibility patterns changed with the introduction of county mass sites and the FEMA community site. The county mass vaccination sites in one county greatly increased accessibilities for populations of minority and poverty. The FEMA community site in the other county accomplished the same. Both the local health department and the federal government played an important role in mitigating pre-existing inequalities during the vaccination campaign. Our study shows that social determinants of health need to be addressed and taken into explicit consideration when planning resource distribution during the pandemic.

## Introduction

The COVID-19 pandemic not only caused profound damage to human health, livelihood of humans and economy worldwide (1), but posed an unprecedented challenge to our healthcare system as well as public health planning and response systems (2). During the early stage of the pandemic, containing the spread and reducing healthcare demand had mostly relied on public health measures known as non-pharmaceutical interventions (NPIs) such as social distancing, contact tracing, travel-related restrictions, and personal protective measures (3). The effectiveness of these interventions, however, has not been consistent across different countries (4, 5) and different states in the US (6) due to a variety of policy and social factors. The fast development and roll-out of COVID-19 vaccines brought a substantial impact on mitigating outbreaks (7) when combined with the NPIs. Mass vaccination offers a crucial pharmaceutical strategy for exiting the pandemic while preventing excessive demands on the health-care system (8).

The United States started its COVID-19 vaccination campaign in December 2020. By spring 2021, vaccine production and supply had increased for vaccination to open up to all adult populations. Counties established community vaccination sites in addition to medical facilities and pharmacies to push for mass vaccinations. The Federal Emergency and Management Agency (FEMA) also established community vaccination centers in dense urban areas (9). Production, accessibility, and acceptance are all essential factors that ensure a majority of the population gets vaccinated at this stage (10). In particular, accessibility to vaccination resources is an important determinant of efficient and equitable vaccine distribution (11).

The existence of disparities in COVID-19 infections and mortality between racial groups in the US has been widely documented (11–14). It calls for actions in various aspects of public health response from data collection to resource allocation in order to avoid further propagating the inequities (12). Examples include establishing testing sites in underserved communities to increase accessibility during the testing and containment stages (14) and incorporating health equity into reopening plans (15). During the current vaccination campaign, health equity should also be addressed through incorporating social factors in the distribution of vaccine resources. Accessibility to vaccine resources by different population groups should be evaluated in a systematic manner across multiple sectors (13) to mitigate disparities and prioritize accessibility of the disproportionately affected racial and ethnic minority groups.

Access to vaccination sites can be evaluated using proximity-based measures such as distance from census tract centroids to vaccination sites (16). Additionally, vaccination capacity of the individual sites affects accessibility as supply varies greatly among sites. Geospatial measures of accessibility take into consideration the spatially varying supply and distribution of the supply to population demands based on travel distance. Previous research measured accessibility to influenza A/H1N1p vaccination sites as vaccination capacity divided by travel distance (17). For each site, accessibility was adjusted by dividing the sum of accessibilities to all nearby sites. It considered other sites as competing factors and the accessibility measure was about one's tendency to get vaccinated at a particular site and not others. We aim to measure one's accessibility to the general vaccine resource. Multiple vaccine sites thus supplement each other and not compete with each other. The study (17) also did not consider the sharing of the vaccine supply among the population. Another study quantified accessibility to H1N1 vaccines by incorporating not only the distance and capacity factors, but also population demand in the service area (18). An optimization approach made it possible to also capture system constraints such as individuals' choices. The optimization model, however, relied on a number of assumptions regarding user choices as well as users' full knowledge of all vaccine sites within 50 miles and their capacities when making their choices. Some of these assumptions could not be met during the COVID-19 vaccination campaign. The model was also computationally intensive and thus best used for retrospective studies and did not fit our goal for rapid assessment of vaccine access among disproportionally affected populations during an ongoing vaccination campaign.

During early stages of the COVID-19 pandemic, studies evaluated accessibilities to testing sites using rapid measures by taking into consideration of testing capacities, population demands, and travel distances (19–21)). We adopted a similar approach to examine the vaccine accessibility landscape in NJ counties surrounding Newark in the NY Metropolitan area. The study area has diverse population makeups and vaccination resources were distributed by a layered system consisting of county mass vaccination sites, medical facilities and pharmacies, as well as a FEMA community vaccination center during the vaccination campaign.

The objectives are to (1) examine the spatial heterogeneity of accessibility in diversely populated communities, (2) investigate the relationship between accessibility and socioeconomic factors, and (3) compare the effect of different types of vaccination sites during the mass vaccination campaign. The goal is to provide insight into any mismatch of resources and population, inform public health planners to guide efforts in establishing sites, and allocate resources in an equitable manner. The method provides rapid assessments with readily available data and is applicable for prospective analysis by public health decision makers during an ongoing vaccination campaign to evaluate scenarios for resource distribution and adjust the setup of vaccination sites. Thus it adds to the toolset for future vaccination planning of other emerging/re-emerging infectious disease outbreaks or if COVID-19 co-exists with the human population for a long time and yearly re-vaccination becomes necessary.

## Methods

This study was performed in Essex County and Union County, New Jersey. New Jersey is one of the most affected US states in the COVID-19 pandemic (40) and the impact has been disproportionately concentrated among Black and Hispanic populations (22, 23). The two counties selected for analysis of vaccine accessibility are located within close proximity to New York City and Newark International Airport, making them a major transmission hub during each wave of the pandemic. Both counties have diverse populations. The largest Essex County racial/ethnic groups are Black (37.5%) followed by White (27.2%) and Hispanic (24.3%). The largest Union County racial/ethnic groups are White (36.7%) followed by Hispanic (33.9%) and Black (19.5%). Both counties started to distribute COVID-19 vaccines through medical facilities and pharmacies and established community vaccination sites in early 2021. One of FEMA's community mass vaccination centers was also located in Newark of Essex County.

### Data

Vaccination site locations as of May 23rd 2021 were obtained through the NJ Department of Health's COVID-19 information hub (24) and geocoded with the ArcGIS World Geocoding Service (25). Daily available appointments at each site were used to represent the vaccination capacity of the individual sites. Socioeconomic data at the census tract level was compiled as Geographic Information System (GIS) maps and associated attribute tables containing variables from the US Census 2019 American Community Survey (ACS), including age and sex, race and ethnicity, income and poverty, housing characteristics, technology and internet availability, among others.

### Accessibility calculation and mapping

We used the two-step floating catchment area (2SFCA) method (26) to calculate accessibility to vaccine resources for each of the 318 census tracts in the two counties. The method computes a supply-to-population ratio to measure accessibility to facilities or resources such as healthcare facilities (27), food resources (28), and most recently COVID-19 testing sites (19) and hospital beds (21). To measure accessibility to vaccines, the first step is to assess the availability of supply at each vaccination site as the ratio of supply to the demand population located within a catchment area of each site (*j*). Delineation of catchment areas is based on a threshold travel distance (*d*_0_) along road networks. A supply-to-demand ratio *R*_*j*_ is then computed for each site using Equation 1.


(1) $$\begin{array}{l} R_{j}=\frac{S_{j}}{\sum_{k\in \left\{d_{kj}\leq d_{0}\right\}}P_{k}} \end{array}$$

*S*_*j*_ is the available vaccination appointments of site *j, P*_*k*_ is the population of census tract *k*, whose centroid falls within the catchment of site *j*. The travel distance *d*_*kj*_ from *k* to *j* is no greater than a preset threshold driving distance *d*_0_. In our study, we tested different thresholds in the delineation of catchments from 1–5 miles. It was observed that with 5-mile catchments, almost all areas in the two counties are covered. As vaccines can be readily administered at many pharmacies, accessibility to vaccination sites should be evaluated differently from accessibility to primary care doctors, for which the commonly used threshold is a 30 min travel time (19). With nearly 9 in 10 Americans living within 5 miles of a community pharmacy (29), we adopted the 5-mile threshold for calculating accessibility scores. One other note in the calculation of accessibility in our study is that the county mass vaccination sites are only open to the county's own residents. Thus we adjusted catchment delineation for county-operated mass sites using county boundaries.

One census tract may fall within the catchments of multiple vaccination sites. The second step of 2SFCA is to calculate the accessibility score *A*_*i*_ for each census tract *i* by summing up the supply of all nearby vaccination sites whose catchment areas contain the census tract *i* using Equation 2, where *A*_*i*_ is the accessibility score calculated for census tract *i*.


(2) $$\begin{array}{l} A_{i}=\sum_{j\in \left\{d_{ij}\leq d_{0}\right\}}R_{j} \end{array}$$

The accessibility scores of the census tracts were mapped with GIS. Cluster and outlier analysis was conducted using the Anselin Local Moran's I (30) to measure the concentration of high and low accessibilities in the study area with ArcGIS Pro (31).

### Accessibility analysis

In order to examine how accessibility varies among different populations, we selected a set of variables from the census attribute table. These include the percentage of population under poverty, percentage of 65 years and older, and percentage of different racial/ethnic groups. We calculated the average accessibility scores of each population group, following Lu et al. (19), using Equation 3. *A*^*g*^ is the average accessibility score of population group *g*, $P_{i}^{g}$ is the percentage of population of group *g* in census tract *i*, and *n* is the total number of census tracts.


(3) $$\begin{array}{l} A^{g}=\frac{\sum_{i=1}^{n}P_{i}^{g}A_{i}}{\sum_{i=1}^{n}P_{i}^{g}} \end{array}$$

In order to test the significance of the differences in accessibility across population groups, we conducted spatial lag regressions with accessibility scores as the dependent variable and the socioeconomic variables as independent variables. A spatial lag of accessibility was added to the linear regression model as an independent variable to account for spatial autocorrelation between neighboring tracts because spatial accessibility measures are usually strongly spatially auto-correlated (19, 26). Specifically, let *y*_*i*_ be the accessibility score of a census tract *i* and the vector of covariates for tract *i* is *x*_*i*_. The model is expressed as:


(4) $$\begin{array}{l} y_{i}=\rho w_{i}y+x_{i}\beta +\varepsilon_{i} \end{array}$$

ε_*i*_ is a random error term. *w*_*i*_*y* is the spatial lag, a weighted average of the spatial neighbors of census tract *i*, defined by a spatial contiguity matrix *W*. ρ represents the relationship between accessibility at a location with accessibilities of its neighbors and βis the vector of local regression coefficients associated with *x*_*i*_. We adopted the first order Queen contiguity to define the spatial contiguity matrix (19, 32) and conducted the analyses using GeoDa 1.18 (33).

In order to examine how a certain socioeconomic factor is correlated with accessibility and how the correlation varies geographically in the two counties, we conducted local bivariate relationship analysis using local entropy maps (34). It allows for the quantification of spatial heterogeneity of the correlation between two variables. It uses a local entropy statistic to measure the amount of shared information between the two involved variables. Entropy can capture complex relationships including exponential, quadratic, and not just linear relationships like other statistics. The results will help us answer specific questions such as: if poverty has a significant correlation with accessibility to vaccination resources, how does the strength of the relationship vary across the different neighborhoods in the two NJ counties? The spatial variation of relationship types and strengths could give insights to resource disparity and guide future public health planning and responses.

## Results

### Spatial heterogeneity of accessibilities

**Figure 1:**
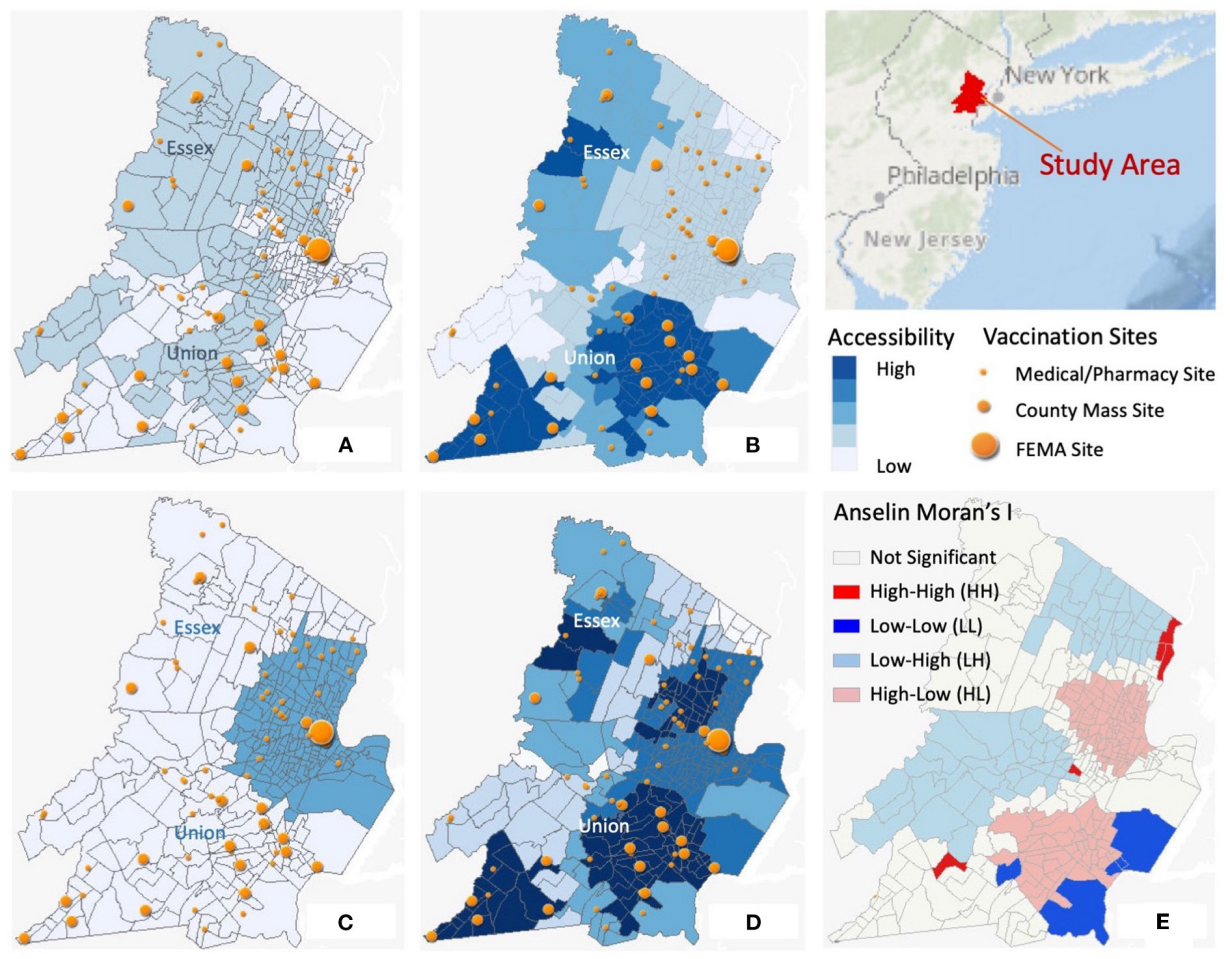
Spatial distributions of accessibility scores. **(A)** Accessibility with medical/pharmacy sites only; **(B)** Accessibility with county mass sites only; **(C)** Accessibility with FEMA sites only; **(D)** Accessibility with all sites combine; **(E)** Cluster analysis with Anselin Moran's I.

Figure 1 shows the distribution of accessibility scores with different types of vaccination sites: medical/pharmacy sites (Figure 1A), county mass vaccination sites (Figure 1B), and FEMA site (Figure 1C). There are notable variations of accessibility depending on the type of facilities. The county sites map visualizes a large cluster in Union county with high accessibility scores compared to the lower accessibilities with only medical/pharmacy sites. In Essex county, high accessibility clusters appear on the northwest side for both county mass sites and medical/pharmacy sites but not the southeast. It is the FEMA community site established in Newark that improved accessibility to the southeast portion of the county. By May 2021, all three types of vaccination sites were in operation. Spatial heterogeneity and local clustering are still notable on the accessibility map combining all vaccine resources (Figure 1D).

The significance of spatial clusters is also confirmed by Anselin Local Moran's I measures. Figure 1E shows significant local clusters include high value clusters (HH) and low value clusters (LL). Significant outliers are neighborhoods with high accessibility surrounded by those with low values (HL), and vice versa (LH). Two small high accessibility score clusters are identifiable in part of Essex (Newark) and Union (Union Township). Two large clusters of low accessibility are located near Montclair in Essex and Springfield in Union. Contrasting accessibility in neighboring communities can be found in many areas such as part of Belleville with high accessibility tracts surrounding low accessibility neighborhoods. These results indicate significant spatial heterogeneity of accessibility to vaccine resources.

### Accessibilities of socioeconomic groups

Table 1 lists the computed average accessibilities of different socioeconomic population groups. In Essex County, Black and Native Americans' accessibilities are lower than average with county mass sites only. So is the accessibility of the population under poverty. Adding medical/pharmacy sites increased overall accessibilities just slightly and did not change the pattern. It was the FEMA site that increased accessibilities greatly and especially for Black, Native, Hispanic populations and the population under poverty. Union county has overall higher accessibilities than Essex. Black and Native Americans' accessibilities are always higher than average starting with county mass sites. So is the accessibility of the population under poverty. Asian and Hispanic populations' accessibilities are slightly lower than average. The FEMA site did not change the situation much as most Union residents live more than 5 miles away from Newark. With all vaccine resources combined, most minority population groups and populations under poverty have higher than average accessibilities in the two counties. Elderly population has slightly lower than average accessibility in both counties. These results indicate that the low accessibility clusters in Union County are not necessarily associated with unfavorable social vulnerability in the aspect of minority population and only corresponds slightly to elderly population.

**Table 1:** Average accessibilities of different population groups.

	**All population**	**White**	**Black**	**Native**	**Asian**	**Hispanic**	**Poverty**	**Elderly**
**Essex county**								
County mass sites	3.15	3.93	2.35	2.12	4.69	3.70	2.33	3.62
Medical/Pharmacy sites	1.11	1.26	1.01	0.88	1.22	1.17	0.95	1.22
County + Medical	4.26	5.19	3.36	3.00	5.92	4.87	3.28	4.84
County + Medical +FEMA	11.6448	9.93	13.29	13.10	10.10	12.41	13.16	11.10
**Union county**								
County mass sites	14.81	12.93	17.53	18.40	11.78	13.16	17.89	13.91
Medical/Pharmacy sites	1.03	1.08	0.97	0.92	1.18	1.06	0.88	1.06
County + Medical	15.84	14.01	18.50	19.31	12.96	14.22	18.77	14.97
County + Medical +FEMA	16.10	14.13	19.22	19.31	13.03	14.71	19.05	15.19

### Spatial lag regression

We fitted spatial lag regression models with two sets of variables to further explore the relationships between the socioeconomic variables and vaccine accessibility. The first set includes the percentage of the total minority population, percentage of population under poverty, percentage of people aged 65 years or older, percentage of the population with no computer and smartphone access, and population density. We conducted spatial lag regression with a second set of variables to break down the percentage of minority population into individual racial/ethnic groups. We performed analyses for accessibility in multiple scenarios: with all vaccination sites combined, with medical sites only, with county mass sites only, and with county mass sites plus medical sites combined. Complete results of this analysis can be found in the Supplementary Material.

The pseudo *R*^2^ values for most spatial lag models range between 0.7 and 0.8 in the various scenarios for the two counties, indicating a moderately strong predictive power. The most significant explanatory variable, however, is the spatial lag variable. This is expected as spatial accessibility measures are often strongly spatially autocorrelated, since neighboring tracts are generally located in close proximity to the same facilities (19). The results show that for Essex County with all vaccination sites combined, most variability of accessibility are explained by such spatial auto-correlation and not most of the other explanatory variables except for the percentage of minority. A positive coefficient, though, suggests a positive correlation, meaning that the higher the minority population, the higher accessibility. This positive relationship is not present with only medical sites, county mass sites, and when the two are combined. Results with the minority population broken down to individual groups show that the percentages of Black and Hispanic populations have significant correlations with accessibility. The correlations, once again, are both positive, indicating the higher these minority population percentages, the higher accessibility to vaccine resources.

In Union County, there is a significant correlation between minority and accessibility and it is also a positive relationship. This relationship starts to be present when we add county mass sites to medical sites. Breaking down to individual minority groups, the significant correlations are present with both Black and Hispanic populations. And similar to Essex county, the correlations are positive, indicating that these minority groups have higher accessibilities.

## Discussion

**Figure 2:**
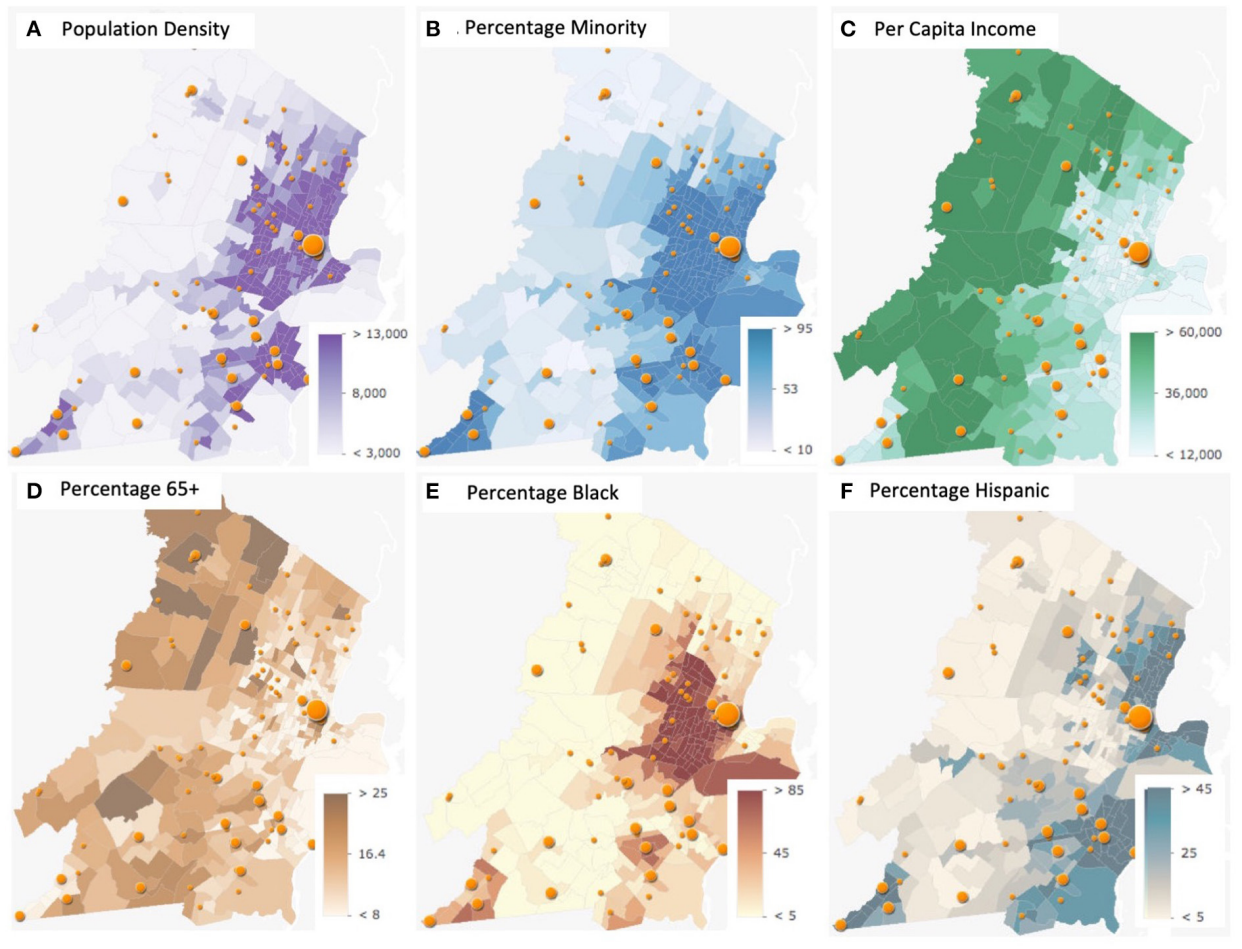
Spatial distributions of socioeconomic variables. **(A)** Population density; **(B)** Percentage minority; **(C)** Per capita income; **(D)** Percentage 65+; **(E)** Percentage black; **(F)** Percentage hispanic.

The results from both Table 1 and spatial lag regressions suggest that minority populations in the two NJ counties do not have disadvantages in their spatial accessibility to vaccine resources, especially with all three types of vaccination sites combined. This can be attributed to the setup of the county mass vaccination sites in Union County and the FEMA community site in Essex County. Figure 2 shows the locations of the different types of vaccination sites overlaid on maps of socioeconomic variables. In Essex County, the county mass sites are more or less evenly distributed over space. Vaccine resources are therefore shared by dense populations in the southeastern region (in and around Newark) with high percentages of poverty and minority populations, resulting in low accessibilities for these populations. The setup of the FEMA site in Newark, supplying 6,000 doses of vaccines every day, corrected this shortage. In Union County, the setup of the county mass sites largely observes the population density patterns, with a number of sites concentrated on the eastern site (Elizabeth) and a small cluster covering the southwestern corner (Plainfield). This setup targets the disproportionately affected populations with high infection and mortality rates, resulting in higher-than-average accessibilities to vaccine resources for such populations.

**Figure 3:**
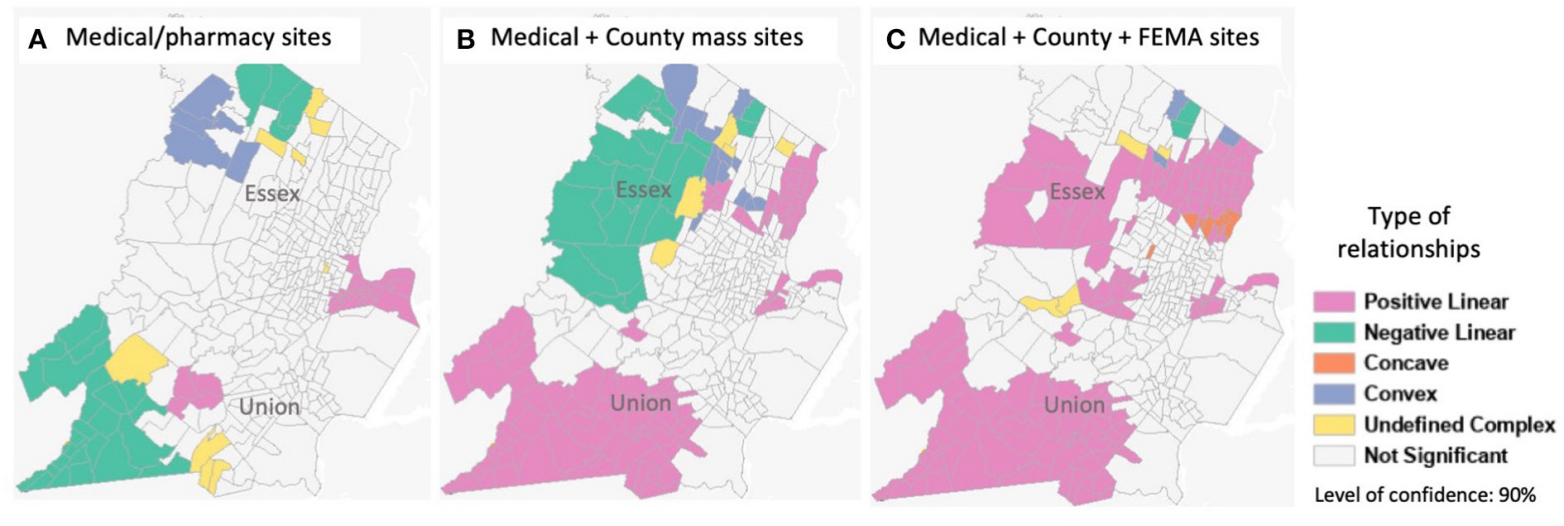
Bivariate relationship analysis between the percentage of minority population and accessibility. **(A)** Medical/pharmacy sites. **(B)** Medical + County mass sites. **(C)** Medical + County + FEMA sites.

Results from the bivariate relationship analysis revealed local correlations between the socioeconomic variables and accessibilities that help us assess the effect of the different vaccination sites. Majority of Essex county did not show significant relationship between the percentage of minority and accessibility with medical sites only (Figure 3A). The introduction of the county mass sites resulted in a large area in the west side having a negative relationship (Figure 3B), that is, lower minority population with higher accessibility.

As shown in Figure 2 and discussed above, this is due to the spatially even distribution of the site locations. The distribution did not consider the greatly varying population density over space and the population distribution of minority groups. The introduction of the FEMA site on the east side corrected this (Figure 3C). In Union County, although a negative correlation was present in the west side of the county with the medical/pharmacy sites, the introduction of county mass sites corrected this, and even resulted in large areas of the county having a positive correlation. That means the higher the minority population, the better accessibility. With all vaccination sites combined (Figure 3C), both counties' minority populations have higher than average accessibilities, confirming the results from Table 1.

### Conclusions

Based on our analyses, a few conclusions can be drawn. First, the COVID-19 pandemic exposed health and socioeconomic inequities in our communities that need to be addressed at the various stages from testing to vaccination. Public health decision making at different levels (county, state, and federal) are all critical in mitigating the disparities. In the case of the two diversely populated New Jersey counties, there was clear spatial heterogeneity in the accessibility to vaccine resources with the existing infrastructure (medical/pharmacy sites). Accessibility patterns changed with the introduction of county mass sites and the FEMA community site. In particular, county mass sites were set up in Union County targeting densely populated areas and minority populations, so was the FEMA community site in Essex County. These resulted in notable changes in the accessibility landscape. It shows how both the local health department and the federal government play an important role in mitigating pre-existing inequalities.

Second, social determinants of health need to be addressed (13) and taken into explicit consideration when distributing resources and evaluating resource accessibility. In our study, the county mass sites in Union County greatly increased accessibilities for populations of minority and poverty. The county mass sites in Essex County, on the other hand, were set up evenly across space, and did not show the same effect. This suggests that the setup of mass vaccination sites should not just cover the geographic space, but need to address the attribute space defined by variables including population density and socioeconomic factors, especially when some socioeconomic groups have been found to be disproportionately affected by the pandemic. Spatial distributions of such socioeconomic variables should direct the vaccine site selections.

Third, utilizing appropriate tools and technology can help improve public health response and decision making. The quick accessibility measure and geospatial analysis used in this study could be applied prospectively during an ongoing mass vaccination campaign to provide essential and timely evaluation of resource accessibility to guide decision making. County health departments can repeatedly run such analyses with various scenarios to guide site selection. Continued analyses could be done to monitor the change of accessibility landscape as vaccine shipments and daily operations of vaccination sites change from time to time.

### Limitations

The accessibility measure used in this study can be quickly calculated with readily available data. Improvements can be made to increase the accuracy of accessibility evaluation, such as considering distance decay (21) or using dynamic catchments (27). Catchment areas could also be delineated based on travel time and transportation modes such as driving, public transportation, and walking could be separately considered. Ecological fallacy is another factor that may affect the accuracy of our accessibility analysis. As we use aggregated data at the census tract level, the inferences about the group may differ from the real experience of individuals (35). Additional analysis is needed to provide more in-depth investigations to individual's experience and perception of accessibility. Additionally, if detailed vaccination record is available, gravity models could be employed to study the flow of population to specific vaccine sites and examine other influencing factors of vaccination such as characteristics of vaccine site locations (17). Lastly, regardless of the setup of the county mass sites and FEMA site prioritizing accessibility for populations disproportionately affected by the pandemic, there is still disparity in vaccination rate among populations and communities. Our study only focused on accessibility to vaccine resources and did not consider one major factor that influences vaccination rates, which is vaccine acceptance/hesitancy (36–39). More in-depth studies on this front could provide valuable insights to innovative solutions in mass vaccination campaigns. The findings could also inform the constraints built in optimization-based methods (18) regarding individual choices.

### Public health implications

This pandemic has shown how marginalized and minority groups have and will suffer disproportionately due to the inequities in society perpetuated by systematic practices. This study is timely to investigate one aspect of health equity, raise consciousness, and use tools and resources to confront inequities. Vaccine distribution system design can greatly influence equity and accessibility at the community level (18). As public health decision makers at different levels set up vaccination sites, evaluating accessibility helps to inform policy and improve coverage and accessibility for disproportionately affected populations. During a vaccination campaign, health equity should be addressed through incorporating social factors in the distribution of vaccine resources to mitigate disparities and prioritize accessibility of the disproportionately affected groups. The results from this study indicate that improving accessibility of these groups can be achieved when site selection considers explicitly the socioeconomic landscape of the population. The methodology employed in this study provides a tool for quick and timely assessment of resource accessibility to make necessary public health decision adjustments during the pandemic.

## Data availability statement

Publicly available datasets were analyzed in this study. This data can be found here: https://covid19.nj.gov/pages/covid-19-vaccine-locations.

## Author contributions

FQ conceived the study and wrote the manuscript. DB and MR implemented the analyses. JL assisted in the analyses and writing. All authors contributed to the article and approved the submitted version.

## Conflict of interest

The authors declare that the research was conducted in the absence of any commercial or financial relationships that could be construed as a potential conflict of interest.

## Publisher's note

All claims expressed in this article are solely those of the authors and do not necessarily represent those of their affiliated organizations, or those of the publisher, the editors and the reviewers. Any product that may be evaluated in this article, or claim that may be made by its manufacturer, is not guaranteed or endorsed by the publisher.
