# Supplementary material for: Evaluating spatial accessibility to COVID-19 vaccine resources in diversely populated counties in the United States
Source: Front Public Health. 2022 Jul 25;10:895538. doi: 10.3389/fpubh.2022.895538 (PMC9358221; doi:10.3389/fpubh.2022.895538)
Supplement: Supplementary file 1 [file Data_Sheet_1.PDF]

Spatial Lag Regression of Accessibility and Socioeconomic Variables
a. Accessibility with all vaccination sites combined

	**Essex County All Sites** (R^2 = 0.71)				**Union County All Sites** (R^2=0.78)			
**Explanatory Variables**	**Coefficient**	**Std. Error**	**z-value**	**p-value**	**Coefficient**	**Std. Error**	**z-value**	**p-value**
Spatial Lag	0.81857	0.04261	19.21040	0	0.77898	0.06123	12.72150	0
Constant	0.00733	0.00379	1.93538	0.05294	-0.00447	0.01012	-0.44222	0.65833
Percentage Minority	2.15E-05	9.61E-06	2.23936	0.02513	7.65E-05	2.22E-05	3.44883	0.00056
Percentage under Poverty	-1.38E-05	2.53E-05	-0.54657	0.58468	-8.22E-05	0.00010	-0.80059	0.42337
Percentage Aged 65+	-5.99E-05	4.33E-05	-1.38202	0.16697	9.21E-05	0.00011	0.86798	0.38540
Population Density	2.24E-09	2.09E-08	0.10757	0.91434	3.59E-08	6.61E-08	0.54214	0.58772
Percent Computer Access	-3.46E-05	4.68E-05	-0.73763	0.46074	-5.53E-05	0.00015	-0.38154	0.70281
Percent Smartphone Access	-2.91E-05	4.24E-05	-0.68739	0.49184	9.33E-05	0.00011	0.86716	0.38586
**Explanatory Variables**	**Coefficient**	**Std. Error**	**z-value**	**p-value**	**Coefficient**	**Std. Error**	**z-value**	**p-value**
Spatial Lag	0.81564	0.04332	18.82830	0	0.79082	0.05999	13.18180	0
Constant	0.00072	0.00092	0.78671	0.43145	-0.00160	0.00238	-0.67250	0.50126
Percentage Under Poverty	-9.07E-06	1.84E-05	-0.49156	0.62303	-8.52E-05	7.13E-05	-1.19502	0.23208
Percentage Aged 65+	-1.88E-05	3.78E-05	-0.49767	0.61872	6.31E-05	1.09E-04	0.57875	0.56276
Percentage Black	2.20E-05	8.69E-06	2.53818	0.01114	6.32E-05	2.15E-05	2.94055	0.00328
Percentage Native American	2.35E-05	2.00E-04	0.11781	0.90622	-3.02E-05	4.81E-04	-0.06277	0.94995
Percentage Asian	3.53E-05	3.60E-05	0.98174	0.32623	1.12E-04	1.09E-04	1.01859	0.30840
Percentage Hawaiian	6.42E-04	9.29E-04	0.69086	0.48965	1.36E-04	2.13E-03	0.06389	0.94906
Percentage Hispanic	2.90E-05	1.20E-05	2.41329	0.01581	9.32E-05	3.28E-05	2.83704	0.00455

Spatial Lag Regression of Accessibility and Socioeconomic Variables
b. Accessibility with medical sites only

	**Essex County Medical Sites** (R^2 = 0.87)				**Union County Medical Sites** (R^2=0.56)			
**Explanatory Variables**	**Coefficient**	**Std. Error**	**z-value**	**p-value**	**Coefficient**	**Std. Error**	**z-value**	**p-value**
Spatial Lag	0.92586	0.02426	38.15750	0	0.74193	0.07232	10.25960	0
Constant	0.00019	0.00032	0.61457	0.53884	5.25E-05	0.00077	0.06834	0.94552
Percentage Minority	-3.09E-07	7.48E-07	-0.41261	0.67989	-8.42E-08	1.55E-06	-0.05443	0.95659
Percentage under Poverty	-8.41E-07	2.12E-06	-0.39753	0.69098	-8.95E-06	7.81E-06	-1.14535	0.25207
Percentage Aged 65+	-2.11E-06	3.59E-06	-0.58816	0.55643	-1.22E-06	8.06E-06	-0.15152	0.87957
Population Density	7.71E-10	1.74E-09	0.44297	0.65779	1.68E-09	5.01E-09	0.33509	0.73756
Percent Computer Access	5.75E-07	3.91E-06	0.14696	0.88316	3.10E-06	1.10E-05	0.28187	0.77805
Percent Smartphone Access	-1.19E-06	3.53E-06	-0.33607	0.73682	-3.40E-07	8.17E-06	-0.04157	0.96684
**Explanatory Variables**	**Coefficient**	**Std. Error**	**z-value**	**p-value**	**Coefficient**	**Std. Error**	**z-value**	**p-value**
Spatial Lag	0.92396	0.02454	37.64480	0	0.72223	0.07545	9.57223	0
Constant	0.00019	8.01E-05	2.38883	0.01690	0.00045	0.00020	2.25290	0.02427
Percentage Under Poverty	-1.65E-06	1.52E-06	-1.08724	0.27693	-7.60E-07	5.35E-06	-0.14194	0.88713
Percentage Aged 65+	-2.32E-06	3.10E-06	-0.74834	0.45425	-7.90E-06	8.18E-06	-0.96582	0.33413
Percentage Black	-4.41E-07	6.68E-07	-0.66079	0.50875	3.39E-07	1.51E-06	0.22445	0.82241
Percentage Native American	3.46E-06	1.64E-05	0.21076	0.83308	1.24E-05	3.60E-05	0.34466	0.73035
Percentage Asian	-3.40E-06	2.95E-06	-1.15214	0.24926	4.32E-06	8.23E-06	0.52461	0.59985
Percentage Hawaiian	0.00012	7.64E-05	1.61558	0.10619	-2.17E-05	1.59E-04	-0.13628	0.89160
Percentage Hispanic	-9.77E-07	9.37E-07	-1.04230	0.29727	-2.66E-06	2.32E-06	-1.14581	0.25187

Spatial Lag Regression of Accessibility and Socioeconomic Variables
c. Accessibility with county mass sites only

	**Essex County Mass Sites** (R^2 = 0.74)				**Union County Mass Sites** (R^2=0.81)			
**Explanatory Variables**	**Coefficient**	**Std. Error**	**z-value**	**p-value**	**Coefficient**	**Std. Error**	**z-value**	**p-value**
Spatial Lag	0.86811	0.03559	24.39510	0	0.80813	0.05562	14.52930	0
Constant	0.00218	0.00239	0.91146	0.36205	-6.68E-05	0.00916	-0.00730	0.99418
Percentage Minority	-4.20E-06	5.73E-06	-0.73274	0.46372	5.90E-05	1.97E-05	2.98715	0.00282
Percentage under Poverty	1.21E-06	1.61E-05	0.07508	0.94015	-4.31E-05	9.28E-05	-0.46447	0.64231
Percentage Aged 65+	6.17E-06	2.73E-05	0.22563	0.82149	8.94E-05	9.59E-05	0.93258	0.35103
Population Density	-1.85E-09	1.32E-08	-0.13991	0.88873	5.14E-08	5.99E-08	0.85736	0.39125
Percent Computer Access	-2.39E-05	2.97E-05	-0.80437	0.42118	-8.66E-05	0.00013	-0.66083	0.50872
Percent Smartphone Access	1.11E-05	2.69E-05	0.41147	0.68073	7.47E-05	9.72E-05	0.76768	0.44268
**Explanatory Variables**	**Coefficient**	**Std. Error**	**z-value**	**p-value**	**Coefficient**	**Std. Error**	**z-value**	**p-value**
Spatial Lag	0.84274	0.03990	21.12150	0	0.82909	0.052621	15.75580	0
Constant	0.00045	0.00054	0.82109	0.41159	-0.00277	0.002114	-1.31034	0.19008
Percentage Under Poverty	3.10E-06	1.10E-05	0.28260	0.77748	-4.88E+00	6.37E-05	-0.76510	0.44421
Percentage Aged 65+	1.42E-05	2.25E-05	0.63070	0.52824	8.50E-05	9.75E-05	0.87192	0.38325
Percentage Black	-4.12E-06	4.86E-06	-0.84738	0.39678	4.94E-05	1.90E-05	2.60348	0.00923
Percentage Native American	-4.21E-05	1.19E-04	-0.35408	0.72328	5.80E-05	4.30E-04	0.13472	0.89284
Percentage Asian	1.15E-05	2.13E-05	0.53884	0.59000	1.53E-04	9.81E-05	1.55666	0.11955
Percentage Hawaiian	7.20E-04	5.53E-04	1.30175	0.19300	3.89E-04	1.90E-03	0.20488	0.83767
Percentage Hispanic	-5.26E-06	6.77E-06	-0.77788	0.43664	8.35E-05	2.94E-05	2.84012	0.00451

Spatial Lag Regression of Accessibility and Socioeconomic Variables
d. Accessibility with county mass sites plus medical sites combined

	**Essex County Mass+Medical Sites** (R^2 = 0.77)				**Union County Mass+Medical Sites** (R^2=0.8)			
**Explanatory Variables**	**Coefficient**	**Std. Error**	**z-value**	**p-value**	**Coefficient**	**Std. Error**	**z-value**	**p-value**
Spatial Lag	0.86811	0.03559	24.39510	0	0.80403	0.05656	14.21680	0
Constant	0.00218	0.00239	0.91146	0.36205	6.33E-05	0.00934	0.00678	0.99459
Percentage Minority	-4.20E-06	5.73E-06	-0.73274	0.46372	5.94E-05	2.01E-05	2.95779	0.00310
Percentage under Poverty	1.21E-06	1.61E-05	0.07508	0.94015	-5.09E-05	9.46E-05	-0.53767	0.59080
Percentage Aged 65+	6.17E-06	2.73E-05	0.22563	0.82149	8.76E-05	9.78E-05	0.89600	0.37025
Population Density	-1.85E-09	1.32E-08	-0.13991	0.88873	5.39E-08	6.11E-08	0.88219	0.37768
Percent Computer Access	-2.39E-05	2.97E-05	-0.80437	0.42118	-8.39E-05	0.00013	-0.62775	0.53017
Percent Smartphone Access	1.11E-05	2.69E-05	0.41147	0.68073	7.33E-05	9.91E-05	0.73921	0.45978
**Explanatory Variables**	**Coefficient**	**Std. Error**	**z-value**	**p-value**	**Coefficient**	**Std. Error**	**z-value**	**p-value**
Spatial Lag	0.86046	0.03686	23.34480	0	0.82637	0.05337	15.48300	0
Constant	0.00067	0.00058	1.14856	0.25074	-0.00242	0.00217	-1.11996	0.26273
Percentage Under Poverty	1.06E-06	1.16E-05	0.09120	0.92733	-4.90E-05	6.51E-05	-0.75276	0.45159
Percentage Aged 65+	1.16E-05	2.38E-05	0.48999	0.62414	7.80E-05	9.95E-05	0.78375	0.43319
Percentage Black	-4.49E-06	5.14E-06	-0.87288	0.38273	5.01E-05	1.94E-05	2.58671	0.00969
Percentage Native American	-3.90E-05	1.26E-04	-0.31025	0.75637	7.02E-05	4.39E-04	0.15998	0.87289
Percentage Asian	5.86E-06	2.25E-05	0.26019	0.79472	1.54E-04	1.00E-04	1.53701	0.12429
Percentage Hawaiian	8.34E-04	5.85E-04	1.42641	0.15375	3.67E-04	1.94E-03	0.18898	0.85011
Percentage Hispanic	-6.20E-06	7.16E-06	-0.86521	0.38693	8.17E-05	2.99E-05	2.73490	0.00624
